# Supplementary figures and images for: Tau and Aβ42 in lavage fluid of pneumonia patients are associated with end-organ dysfunction: A prospective exploratory study
Source: PLoS One. 2024 Feb 23;19(2):e0298816. doi: 10.1371/journal.pone.0298816 (PMC10889620; doi:10.1371/journal.pone.0298816)

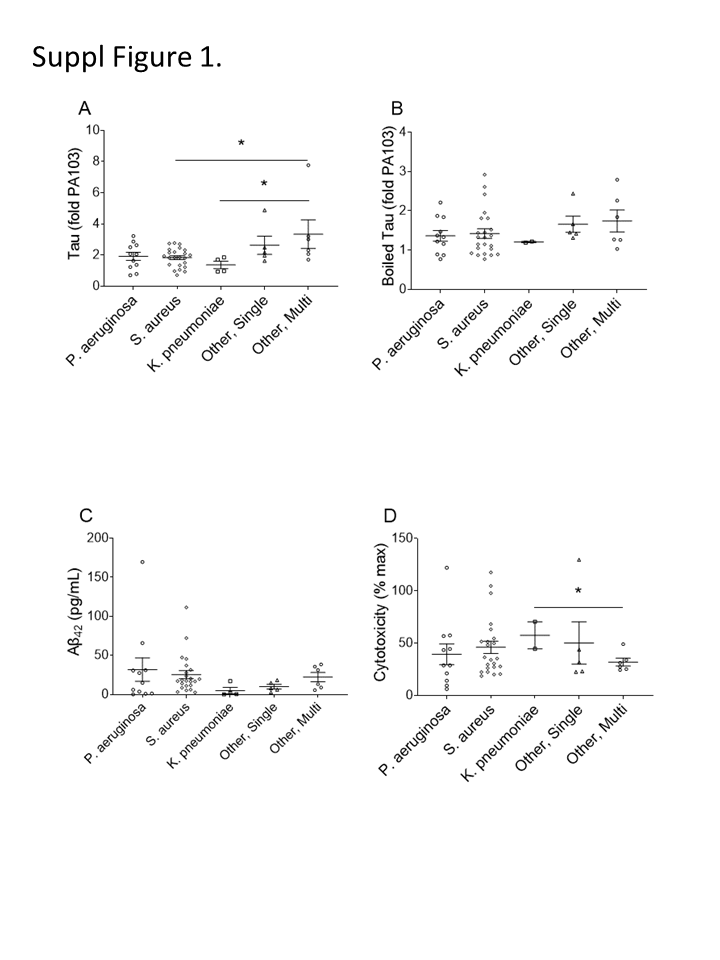

Supplement: S1 Fig — (A) Tau, (B) boiled tau, (C) Aβ42, and (D) cytotoxicity were assayed as described in the Methods in different species of bacteria: Pseudomonas aeruginosa, Staphylococcus aureus, Klebsiella pneumoniae, other single isolates, and other multi-microbial isolates. Data are expressed as mean ± SEM, n are listed below groups; * represents p < 0.05. (TIF) [file pone.0298816.s001.tif]

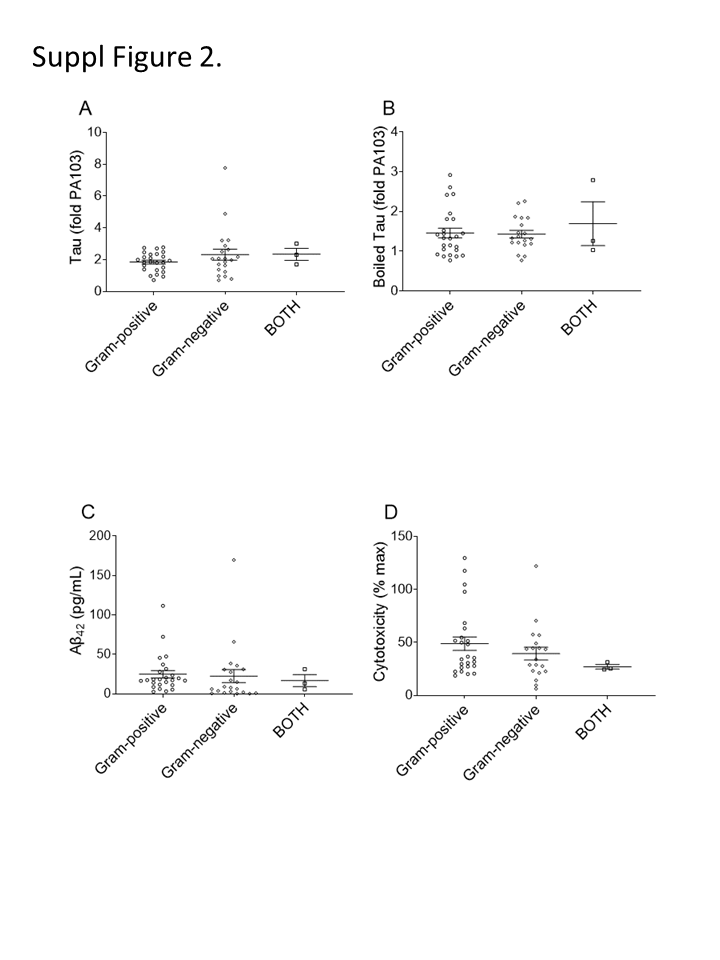

Supplement: S2 Fig — (A) Tau, (B) boiled tau, (C) Aβ42, and (D) cytotoxicity were assayed as described in the Methods in different phenotypes of bacteria: Gram-positive, Gram-negative, or both. Data are expressed as mean ± SEM, n are listed below groups. (TIF) [file pone.0298816.s002.tif]
