# Supplementary material for: Tau and Aβ42 in lavage fluid of pneumonia patients are associated with end-organ dysfunction: A prospective exploratory study
Source: PLoS One. 2024 Feb 23;19(2):e0298816. doi: 10.1371/journal.pone.0298816 (PMC10889620; doi:10.1371/journal.pone.0298816)
Supplement: Attachment — Submitted filename: Response to Reviewers.docx [file pone.0298816.s004.docx]

Dear Dr. Ginsberg and Reviewers,

Thank you for your insightful review of our manuscript. We appreciate your time and effort in making our work the best it can be. Below, please find the original comments/questions from the journal, editor, and reviewers and our responses, edits, additions, etc.

We have included “clean” and “marked” copies of the manuscript as requested.

**Journal Requirements:**

1. When submitting your revision, we need you to address these additional requirements.

Please ensure that your manuscript meets PLOS ONE's style requirements, including those for file naming. The PLOS ONE style templates can be found at [https://journals.plos.org/plosone/s/file?id=wjVg/PLOSOne_formatting_sample_main_body.pdf](https://urldefense.com/v3/__https:/journals.plos.org/plosone/s/file?id=wjVg*PLOSOne_formatting_sample_main_body.pdf__;Lw!!NoSwA-eRAg!FJoUnZ5j-7jfJBf21h9eWQ1Sl5CG6q16aw2KODIORokjAYimhhTEWaCo15QsA5fsNWSrFEa8awMlxcKwtg$) and <https://journals.plos.org/plosone/s/file?id=ba62/PLOSOne_formatting_sample_title_authors_affiliations.pdf>

**Response**: We appreciate the journal’s commitment to quality. We have reviewed the instructions provided, including the two template files, and have done our best to apply this formatting style to our manuscript, including file naming.

2. Thank you for stating in your Funding Statement:

This study was supported by:

B.M.W.--GM127584 and GM127584-S1; National Institute of General Medical Sciences; [https://www.nigms.nih.gov](https://urldefense.com/v3/__https:/www.nigms.nih.gov__;!!NoSwA-eRAg!FJoUnZ5j-7jfJBf21h9eWQ1Sl5CG6q16aw2KODIORokjAYimhhTEWaCo15QsA5fsNWSrFEa8awOd51JBeQ$)

T. S. and R.B.--HL66299 and HL148069; National Heart, Lung, and Blood Institute; [https://www.nhlbi.nih.gov](https://urldefense.com/v3/__https:/www.nhlbi.nih.gov__;!!NoSwA-eRAg!FJoUnZ5j-7jfJBf21h9eWQ1Sl5CG6q16aw2KODIORokjAYimhhTEWaCo15QsA5fsNWSrFEa8awM8HMXkug$)

M.L., T.S., R.B.--HL140182; National Heart, Lung, and Blood Institute; [https://www.nhlbi.nih.gov](https://urldefense.com/v3/__https:/www.nhlbi.nih.gov__;!!NoSwA-eRAg!FJoUnZ5j-7jfJBf21h9eWQ1Sl5CG6q16aw2KODIORokjAYimhhTEWaCo15QsA5fsNWSrFEa8awM8HMXkug$)

J.P.A. and D.A.--HL118334; National Heart, Lung, and Blood Institute; [https://www.nhlbi.nih.gov](https://urldefense.com/v3/__https:/www.nhlbi.nih.gov__;!!NoSwA-eRAg!FJoUnZ5j-7jfJBf21h9eWQ1Sl5CG6q16aw2KODIORokjAYimhhTEWaCo15QsA5fsNWSrFEa8awM8HMXkug$)

S.M.L--AI170719; National Institute of Allergy and Infectious Diseases; [https://www.niaid.nih.gov](https://urldefense.com/v3/__https:/www.niaid.nih.gov__;!!NoSwA-eRAg!FJoUnZ5j-7jfJBf21h9eWQ1Sl5CG6q16aw2KODIORokjAYimhhTEWaCo15QsA5fsNWSrFEa8awO0oAktyw$)

S.V.--HL147512, HL007778, REAP220049A0001; National Heart, Lung, and Blood Institute; [https://www.nhlbi.nih.gov](https://urldefense.com/v3/__https:/www.nhlbi.nih.gov__;!!NoSwA-eRAg!FJoUnZ5j-7jfJBf21h9eWQ1Sl5CG6q16aw2KODIORokjAYimhhTEWaCo15QsA5fsNWSrFEa8awM8HMXkug$) and Edward Via College of Osteopathic Medicine Research Eureka Accelerator Program (REAP); [https://www.vcom.edu/research/research-strategic-plan](https://urldefense.com/v3/__https:/www.vcom.edu/research/research-strategic-plan__;!!NoSwA-eRAg!FJoUnZ5j-7jfJBf21h9eWQ1Sl5CG6q16aw2KODIORokjAYimhhTEWaCo15QsA5fsNWSrFEa8awNHt9Iddw$)

None of the sponsors or funders played any role in the study design, data collection and analysis, decision to publish, or preparation of the manuscript.

Please provide an amended statement that declares all the funding or sources of support (whether external or internal to your organization) received during this study, as detailed online in our guide for authors at [http://journals.plos.org/plosone/s/submit-now.](https://urldefense.com/v3/__http:/journals.plos.org/plosone/s/submit-now.__;!!NoSwA-eRAg!FJoUnZ5j-7jfJBf21h9eWQ1Sl5CG6q16aw2KODIORokjAYimhhTEWaCo15QsA5fsNWSrFEa8awNDGbHyNw$)  Please also include the statement “There was no additional external funding received for this study.” in your updated Funding Statement.

Please include your amended Funding Statement within your cover letter. We will change the online submission form on your behalf.

**Response**: We appreciate the journal’s commitment to quality. We have amended our statement per the journal guidelines and have added the extra statement as requested. The revised statement can be found in the Cover Letter as requested.

3. In your Data Availability statement, you have not specified where the minimal data set underlying the results described in your manuscript can be found. PLOS defines a study's minimal data set as the underlying data used to reach the conclusions drawn in the manuscript and any additional data required to replicate the reported study findings in their entirety. All PLOS journals require that the minimal data set be made fully available. For more information about our data policy, please see [http://journals.plos.org/plosone/s/data-availability](https://urldefense.com/v3/__http:/journals.plos.org/plosone/s/data-availability__;!!NoSwA-eRAg!FJoUnZ5j-7jfJBf21h9eWQ1Sl5CG6q16aw2KODIORokjAYimhhTEWaCo15QsA5fsNWSrFEa8awMes5F0nw$).

Upon re-submitting your revised manuscript, please upload your study’s minimal underlying data set as either Supporting Information files or to a stable, public repository and include the relevant URLs, DOIs, or accession numbers within your revised cover letter. For a list of acceptable repositories, please see [http://journals.plos.org/plosone/s/data-availability#loc-recommended-repositories](https://urldefense.com/v3/__http:/journals.plos.org/plosone/s/data-availability*loc-recommended-repositories__;Iw!!NoSwA-eRAg!FJoUnZ5j-7jfJBf21h9eWQ1Sl5CG6q16aw2KODIORokjAYimhhTEWaCo15QsA5fsNWSrFEa8awPSEuhRtg$). Any potentially identifying patient information must be fully anonymized.

Important: If there are ethical or legal restrictions to sharing your data publicly, please explain these restrictions in detail. Please see our guidelines for more information on what we consider unacceptable restrictions to publicly sharing data: [http://journals.plos.org/plosone/s/data-availability#loc-unacceptable-data-access-restrictions](https://urldefense.com/v3/__http:/journals.plos.org/plosone/s/data-availability*loc-unacceptable-data-access-restrictions__;Iw!!NoSwA-eRAg!FJoUnZ5j-7jfJBf21h9eWQ1Sl5CG6q16aw2KODIORokjAYimhhTEWaCo15QsA5fsNWSrFEa8awO-uZ4IZA$). Note that it is not acceptable for the authors to be the sole named individuals responsible for ensuring data access.

We will update your Data Availability statement to reflect the information you provide in your cover letter.

**Response**: We appreciate the journal’s commitment to quality. We have added our minimal data set as a Supporting Information file as requested. We have added this as a supporting information file entitled “S1_Minimal Data Set” and updated our Data Availability statement in the Cover Letter, as requested.

4. Please review your reference list to ensure that it is complete and correct. If you have cited papers that have been retracted, please include the rationale for doing so in the manuscript text, or remove these references and replace them with relevant current references. Any changes to the reference list should be mentioned in the rebuttal letter that accompanies your revised manuscript. If you need to cite a retracted article, indicate the article’s retracted status in the References list and also include a citation and full reference for the retraction notice.

**Response**: We appreciate the journal’s commitment to quality. We have reviewed our reference list as requested.

**Additional Editor Comments**:

Thank you for submitting your work to PLOS ONE. After careful consideration by 2 Reviewers and an Academic Editor, please make the corrections posed by Reviewers #1 and #2 so I can render a decision on this manuscript.

**Response**: We thank the Editor for their comments and appreciate his time and effort. We will respond to the comments accordingly.

**Comments to the Author**

1. Is the manuscript technically sound, and do the data support the conclusions?

The manuscript must describe a technically sound piece of scientific research with data that supports the conclusions. Experiments must have been conducted rigorously, with appropriate controls, replication, and sample sizes. The conclusions must be drawn appropriately based on the data presented.

Reviewer #1: Yes

Reviewer #2: Yes

**Response**: We thank the reviewers for their comments and appreciate their time and effort.

2. Has the statistical analysis been performed appropriately and rigorously?

Reviewer #1: Yes

Reviewer #2: Yes

**Response**: We thank the reviewers for their comments and appreciate their time and effort.

3. Have the authors made all data underlying the findings in their manuscript fully available?

The [PLOS Data policy](https://urldefense.com/v3/__http:/www.plosone.org/static/policies.action*sharing__;Iw!!NoSwA-eRAg!FJoUnZ5j-7jfJBf21h9eWQ1Sl5CG6q16aw2KODIORokjAYimhhTEWaCo15QsA5fsNWSrFEa8awNK-26ONQ$) requires authors to make all data underlying the findings described in their manuscript fully available without restriction, with rare exception (please refer to the Data Availability Statement in the manuscript PDF file). The data should be provided as part of the manuscript or its supporting information, or deposited to a public repository. For example, in addition to summary statistics, the data points behind means, medians and variance measures should be available. If there are restrictions on publicly sharing data—e.g. participant privacy or use of data from a third party—those must be specified.

Reviewer #1: Yes

Reviewer #2: No

**Response**: We thank the reviewers for their comments and appreciate their time and effort. See our response above. We are submitting our minimal data set as a Supporting Information file.

4. Is the manuscript presented in an intelligible fashion and written in standard English?

PLOS ONE does not copyedit accepted manuscripts, so the language in submitted articles must be clear, correct, and unambiguous. Any typographical or grammatical errors should be corrected at revision, so please note any specific errors here.

Reviewer #1: Yes

Reviewer #2: Yes

**Response**: We thank the reviewers for their comments and appreciate their time and effort.

5. Review Comments to the Author

**Reviewer #1**: Overall the scientific premise of the study is interesting, and the use of the patients from four intensive care units to address the hypothesis is appropriate. The references are appropriate and most recent. However, the study is required some refinement as follows.

**Response**: We thank the reviewers for their positive comments and appreciate their time and effort.

Comment1: The abstract is well written, but I would like to see the number of patients included in Bacterial culture-positive and culture-negative groups as well as mean age and % of males in both groups in the abstract.

**Response**: We thank the reviewer for this comment. We have modified the Abstract as requested.

Comment2: In Introduction, provide the reference that describe the involvement of amyloid and tau in Parkinson's disease.

Also change Picks disease to frontotemporal tauopathies.

**Response**: We thank the reviewer for this comment. We have added two references as requested (see below for details) and have changed “Pick’s Disease” to “frontotemporal tauopathies”.

Kang JH, Irwin DJ, Chen-Plotkin AS, Siderowf A, Caspell C, Coffey CS, et al. Association of cerebrospinal fluid beta-amyloid 1-42, T-tau, P-tau181, and alpha-synuclein levels with clinical features of drug-naive patients with early Parkinson disease. JAMA Neurol. 2013;70(10):1277-87.

Irwin DJ, Lee VM, Trojanowski JQ. Parkinson's disease dementia: convergence of alpha-synuclein, tau and amyloid-beta pathologies. Nat Rev Neurosci. 2013;14(9):626-36.

Comment3: There was difference in cytotoxic tau and amyloids levels by race which is interesting. I would also like to present this novel finding in the abstract as well as discuss in the discussion.

**Response**: We thank the reviewer for this comment. We agree that this is interesting and have added this finding to the abstract and have discussed this particular topic, as requested.

Specifically, for the Discussion, we added:

Finally, tau, boiled tau, and Aβ42 were increased in Caucasian CP patients compared to both Caucasian and minority CN patients. The difference between Caucasian CP and CN patients is consistent with our initial hypothesis that tau, boiled tau, and Aβ42 and lead to end-organ dysfunction. However, the increases in tau, boiled tau, and Aβ42 between Caucasian CP and minority CN groups is less clear. While there may be social, demographic, regional, or generic differences between these groups that lead to differential susceptibility to bacterial pneumonia, this does not cover one important negative finding—there is no difference in tau, boiled tau, and Aβ42 between minority CP and CN patients. The baseline levels of tau, boiled tau, and Aβ42 in minority CN patients is similar to Caucasian CN patients; however, there is no significant increase in tau, boiled tau, and Aβ42 when minority patients are infected with bacterial pneumonia. It is possible that minority patients do not elicit tau, boiled tau, or Aβ42 after bacterial pneumonia for a variety and these possibilities should be explored. However, large population studies indicate that minority patients have delayed diagnosis of sepsis, of which bacterial pneumonia is a source (*, ref). It is possible that tau, boiled tau, and Aβ42 increase and then decrease within a short time frame and, if care providers are delayed in diagnosing bacterial pneumonia, the increase seen in Caucasian CP patients might be missed.

Comment 3: In table 1, add the SD for age in total patients and well as add the percent of male in total patients' column.

**Response**: We thank the reviewer for this comment. We have added the changes to Table I as requested by the reviewer.

Comment 4: How data of smoking, Alcohol and Illicit Drug collected? were they self-reported?

**Response**: We thank the reviewer for this comment. These items were collected from ICU admission notes for each patient. These data are self-reported or reported by family.

Comment 5: Is the lung injury associated with amyloid and tau in CP group also common in male than females?

**Response**: We thank the reviewer for this comment. We agree that sex dimorphism in a variety of research areas, including bacterial pneumonia and chronic critical illness, is of great interest. We analyzed the groups requested by the reviewer; however, there were no significant differences between male/female LIS in amyloid/tau/boiled tau responders. The data below has not been added to the manuscript, but is being provided to the reviewer for their benefit.

Male LIS (mean ± SD; n)	Female LIS (mean ± SD; n)

Aβ_42_	1.23 ± 0.68 (17)	1.23 ± 0.67 (8)

Tau	1.13 ± 0.65 (29)	1.38 ± 0.34 (13)

Boiled Tau	1.20 ± 0.66 (26)	1.28 ± 0.37 (12)

Comment 6: the cytotoxicity is more in female CP patients compared to both male and female CN patients even after male CP exhibited more amyloid and tau level than female CP. this is in contrast. I would suggest discussing in the discussion.

**Response**: We thank the reviewer for this comment. We agree that this is an intriguing difference and have broadened our discussion of this topic, as requested. Edits have been made in various areas around lines 365-374.

**Reviewer #2**:

Introduction –

Two fundamental questions remain: how do A-beta and p-tau produced in the lungs reach the brain? The BBB is impermeable to macromolecular compounds. A-beta in the brain is deposited intravascular or in the parenchyma and NFT in the nerve cell. Moreover, compounds present in the cerebrospinal fluid reflect central pathology. It is also known that pathological changes in AD precede central pathology for many years and begin with the deposition of pathological proteins in the heart and other organs. I don't know if the authors made good research assumptions.

Hypotheses of the AD pathophysiology include amyloid cascade, inflammation, vascular, and infection factors. e.g. Grobler et al. PMID: 36442193

**Response**: We really appreciate the points made here by the reviewer, and we have considered them extensively as our work has been progressing. As pointed out, much of what we know about phospho-tau and beta amyloid comes from studies of chronic neurodegenerative diseases. Based on this extensive body of work, the field has come to appreciate the points highlighted here: (1) that beta amyloid in the brain is deposited intravascular or in the parenchyma; (2) that phospho-tau is assembled in neurofibrillary tangles in neurons; (3) that compounds present in the cerebrospinal fluid reflect central pathology; and, (4) that pathological changes in chronic dementias precede central pathology for many years and begin with the deposition of pathological proteins in the heart and other organs. While these findings are all very consistent in Alzheimer’s disease and related pathologies, this is not the case with acute onset of infectious proteinopathy, as occurs following pneumonia.

To that end, we and other investigators have discovered non-neuronal expression and function of tau and amyloid precursor protein/beta-amyloid, in what some investigators refer to as “peripheral amyloids” (for review see *Front. Aging Neurosci.,* 14: 858429, 2022). More specifically, we have seen that tau and amyloid precursor protein are expressed in the lung, especially in lung capillary endothelium. We have recently reviewed the work that has led up to our current understanding of the lung capillary endothelial cell expression of tau and amyloid precursor protein in *Physiol. Rev.,* 104: 533-649, 2024. Much of the normal physiological function of tau and beta amyloid in the lung microcirculation remains poorly understood, yet available data suggests tau contributes to microtubule architecture and barrier integrity whereas constitutive beta amyloid generation seems to have antimicrobial properties. Studies are ongoing to better resolve the physiological importance of tau and beta amyloid production in the lung parenchyma.

However, it is evident that infection of the lower airways leads to generation of cytopathic variants of tau and beta amyloid within the lung. This assertion is based on several preclinical studies as discussed in the paper, and now, more comprehensive assessment of bronchoalveolar lavage fluid from a prospective study of infected versus uninfected patients. This phenomenon is different than what has been described in the neurosciences. It is most related to infection and critical care medicine.

Our preclinical studies provide compelling evidence that during and following lower airway infection, cytopathic forms of tau and beta amyloid are retrieved from the bronchoalveolar lavage fluid, the blood, and the blood parenchyma. We have consistently seen that within 48 hours of infection, cytopathic amyloid variants can be recovered from the brain parenchyma. The reviewer points out that it is unclear how they could access the brain. We agree with this assertion. At present, we do not know how the cytopathic species disseminate into the brain. We have considered several possibilities. Sepsis on the systemic side does lead to neurologic damage without direct brain injury (no traumatic brain injury or stroke) *Neurol Res.,* 46:195-206, 2024. Alternatively, they may be transported across the blood brain barrier, or perhaps via the choroid plexus. Furthermore, there are other access points to the cerebrospinal fluid, and the brain, that are independent of the BBB directly, such as the glymphatic system *Physiol. Rev.,* 104: 533-649, 2024. These possibilities have been discussed in our recent review, but they remain active areas of study.

Our understanding of this infectious proteinopathy is growing, but we are aware that there remains a lot to learn. We have been careful to conservatively interpret our findings, and in this paper in particular, we have not made assertions about the whether or not tau and beta amyloid in the bronchoalveolar lavage fluid are associated with cognitive dysfunction, i.e., we did not measure cognitive dysfunction in this paper. Nonetheless, in this study, our hypothesis, data, results, and discussion all support the idea that increased tau and beta amyloid after bacterial pneumonia are associated with end-organ dysfunction, all found in the systemic side of the blood brain barrier. Studies addressing the relationship between tau and beta amyloid in the airways following infection and cognitive dysfunction will be undertaken in forthcoming studies.

The reviewers reference was added to our paper and is Reference #27.

Conclusion-

The use of A-beta and p-tau for treatment seems unattainable in the context of incurable neurodegenerative diseases.

**Response**: We thank the reviewer for this comment. We agree that treatment of neurodegenerative diseases at this point in time seems difficult, if not unattainable. However, treatment of cognitive dysfunction is not the goal of our study. We did mention that therapies may arise from our findings, we were not specific to the brain. We demonstrate that increased amyloids/tau after bacterial pneumonia are associated with end-organ dysfunction in other systemic organs. Also, diabetes, and other chronic maladies have been associated with amyloidopathies *J Alzheimers Dis Rep*., 6:349-57, 2022. This study may shed light on new sources of cytotoxic amyloids/tau that may improve pathophysiologic understanding of chronic critical illness. Given our limited understanding of these new-found phenomena, many potential therapies (directed at amyloid/tau) or not, are possible. They may not even need to cross the BBB to be effective.

Improve editorial errors.

**Response**: We thank the reviewer for this comment. We have done our best to spell-check, improve grammar/syntax errors to improve the readability of our manuscript.

Again, we appreciate the reviewer’s insight and assistance to improve our manuscript and make it more readable, understandable, and scientifically rigorous for *PLOS One*.

We hope that our responses satisfy the reviewer’s concerns and we again thank the reviewers for comments that add clarity and significance to our manuscript.

Warm Regards,

Brant M. Wagener, MD, PhD
